# Prefrontal Norepinephrine Determines Attribution of “High” Motivational Salience

**DOI:** 10.1371/journal.pone.0003044

**Published:** 2008-08-22

**Authors:** Rossella Ventura, Emanuele Claudio Latagliata, Cristina Morrone, Immacolata La Mela, Stefano Puglisi-Allegra

**Affiliations:** 1 Santa Lucia Foundation, European Centre for Brain Research (CERC), Rome, Italy; 2 Dipartimento di Scienze e Tecnologie Biomediche, Università dell' Aquila, Aquila, Italy; 3 Dipartimento di Psicologia and Centro “Daniel Bovet”, University “La Sapienza”, Rome, Italy; Victoria University of Wellington, New Zealand

## Abstract

Intense motivational salience attribution is considered to have a major role in the development of different psychopathologies. Numerous brain areas are involved in “normal” motivational salience attribution processes; however, it is not clear whether common or different neural mechanisms also underlie intense motivational salience attribution. To elucidate this a brain area and a neural system had to be envisaged that were involved only in motivational salience attribution to highly salient stimuli. Using intracerebral microdialysis, we found that natural stimuli induced an increase in norepinephrine release in the medial prefrontal cortex of mice proportional to their salience, and that selective prefrontal norepinephrine depletion abolished the increase of norepinephrine release in the medial prefrontal cortex induced by exposure to appetitive (palatable food) or aversive (light) stimuli independently of salience. However, selective norepinephrine depletion in the medial prefrontal cortex impaired the place conditioning induced exclusively by highly salient stimuli, thus indicating that prefrontal noradrenergic transmission determines approach or avoidance responses to both reward- and aversion-related natural stimuli only when the salience of the unconditioned natural stimulus is high enough to induce sustained norepinephrine outflow. This affirms that prefrontal noradrenergic transmission determines motivational salience attribution selectively when intense motivational salience is processed, as in conditions that characterize psychopathological outcomes.

## Introduction

Different neuropsychiatric disorders, such as drug addiction, schizophrenia, attention deficit hyperactivity, anxiety disorders, food disorders and depression, have been related to a breakdown of proper salience assignment [1]–[6].

Prefrontal cortex regulates overall motivational salience and determines the intensity of behavioral responding [7]–[9]. Increased prefrontal norepinephrine (NE) outflow has been reported in response to both rewarding and aversive unconditioned stimuli with high motivational salience [10]–[17].

Appetitive stimuli have been shown to influence noradrenergic activation in prefrontal cortex in a graded manner depending on the salience [15], [16]. However, to our knowledge no evidence exists proving graded prefrontal cortical NE release induced also by aversive stimuli or, most importantly, the functional role of salience-dependent graded prefrontal NE outflow for behavioral outcomes. Here we hypothesized that also aversive stimuli produce a graded activation of prefrontal noradrenergic transmission.

First, using mice, we set out to assess whether the salience of unconditioned natural stimuli, modulated by their intrinsic properties as well as by the motivational state of the organism, is related to increased prefrontal NE outflow regardless of valence, in order to evaluate if prefrontal NE release could be considered an index of stimuli salience.

Increased prefrontal NE outflow is considered to be involved in processing highly arousing unconditioned stimuli and a functional role of prefrontal NE transmission has been proposed to be necessary only under certain conditions, as when animals are taxed by increasing exogenous or endogenous arousal [18]–[20]. On this basis, we hypothesized that prefrontal cortical NE transmission is engaged in motivated behavior only when highly arousing, salient stimuli have to be processed. In a second series of experiments, we assessed the effects of selective prefrontal cortical NE depletion on conditioned place preference (CPP) and on conditioned place aversion (CPA) induced by rewarding and aversive natural stimuli respectively.

A place conditioning procedure was chosen for this study because it permits assessing the acquisition of conditioned appetitive and aversive properties to stimuli paired with primary rewards and aversive events and because a large body of evidence shows that it is a reliable measure of processes underlying motivational salience attribution to stimuli [21], [22]. The salience of stimuli was graded by modulating either their intrinsic properties (highly or mildly salient stimulus) or the motivational state of the organism (control or stressed animals), in the latter case affecting “perceived” salience.

In conclusion, we hypothesized that NE in medial prefrontal cortex (mpFC) is a neural system sensitive to the affective intensity of stimuli, irrespectively of valence and that differently salient stimuli induce a graded activation of prefrontal noradrenergic transmission engaging it in motivational salience attribution only when stronger activation is induced by highly salient stimuli.

Here we found that natural stimuli induced an increase in norepinephrine release in the medial prefrontal cortex of mice proportional to their salience. However, selective norepinephrine depletion in the medial prefrontal cortex impaired the place conditioning induced exclusively by highly salient stimuli, thus indicating that prefrontal noradrenergic transmission determines attribution of motivational salience to both reward- and aversion-related natural stimuli only when the salience of the unconditioned natural stimulus is high enough to induce sustained norepinephrine outflow.

## Results

### Rewarding and aversive stimuli

Both rewarding and aversive stimuli were chosen in order to grade their salience by modulating their intrinsic properties (highly or mildly salient stimulus). Natural stimuli used in this study were the following: white chocolate (WCh, highly salient) or milk chocolate (MCh, mildly salient) as rewarding natural stimuli, and intermittent pulsating light (IPL, highly salient) or intermittent light (IL, mildly salient) as aversive natural stimuli. Milk chocolate (Milka, Kraft) was chosen based on a previous study showing its rewarding properties [17]. White chocolate (Milka, Kraft) was chosen based on a preliminary experiment indicating stronger preference in comparison with the other rewarding stimulus used here, that is milk chocolate, in a conditioned place preference (CPP) test (see Materials and Methods). In fact, behavioral testing revealed a significant preference for the white chocolate-paired chamber (Paired) compared with the milk chocolate-paired chamber (Unpaired) (repeated measure ANOVA: (F (1,14) = 5.8; p<0.05) (Paired: 504±18; Unpaired: 367±12), thus indicating that white chocolate was preferred to milk chocolate in the place conditioning procedure. Intermittent and intermittent pulsating lights were chosen based on previous studies showing their aversive properties [23]. Moreover, a preliminary experiment indicated a stronger aversion for intermittent pulsating light than for intermittent light in a conditioned place aversion (CPA) test (see Materials and Methods). In fact, behavioral testing revealed a significant aversion for the intermittent pulsating light-paired chamber (Paired) compared with the intermittent light-paired chamber (Unpaired) (repeated measure ANOVA: (F (1,14) = 5.74; p<0.05) (Paired: 409±18; Unpaired: 503±34), thus indicating that intermittent pulsating light was more aversive than intermittent light in the place conditioning procedure.

Finally, the perceived salience of mildly salient stimuli (milk chocolate, intermittent light) has been increased by modulating the motivational state of the organism through a chronic stressful condition, since it is known that chronic stress enhances the salience of stimuli [2], [24]. Mice were assigned to different feeding regimen, namely either receiving food ad libitum (Controls) or subjected to food-restriction regimen (Stressed, (FR)).

### Effects of graded salience on prefrontal norepinephrine

Because, prior experience is a major determinant of the motivational impact of any given stimulus [25], we assessed the effects of first exposure to appetitive and aversive stimuli.

We hypothesized that both appetitive and aversive stimuli produce a graded activation of prefrontal noradrenergic transmission, thus the more salient a stimulus the stronger prefrontal NE release will be. If this is the case, prefrontal NE release could be considered an index of stimuli salience. We used intracerebral microdialysis to evaluate the effects of the first exposure to different rewarding or aversive graded salient natural stimuli on prefrontal cortical NE release of Sham animals. Moreover, we evaluated whether selective prefrontal NE depletion impairs the increase in prefrontal cortical NE release induced by these stimuli. Selective depletion of prefrontal cortical NE afferents (NE depleted groups) produced a profound depletion of tissue levels of norepinephrine (∼90%), leaving tissue levels of dopamine virtually unaffected (see Materials and Methods).

**Figure 1:**
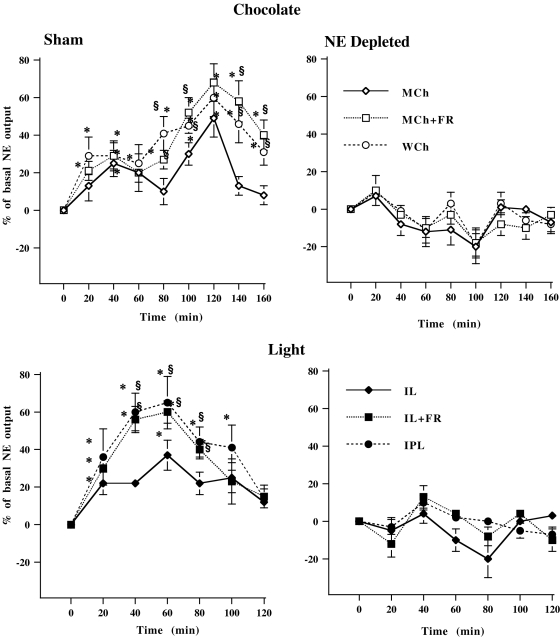
Prefrontal cortical norepinephrine depletion on extracellular norepinephrine in mpFC. Extracellular norepinephrine (NE) in mpFC of Sham or norepinephrine-depleteted (NE Depleted) animals exposed to chocolate (1 g) or light (milk chocolate in control, MCh; milk chocolate in food restricted, MCh+FR; white chocolate in control, WCh; intermittent light in control, IL; intermittent light in food restricted, IL+FR; intermittent-pulsating light in control, IPL). Results are expressed as percent changes from basal levels of each experimental group (*n* = 6–9 per group). Statistical analyses were carried out on raw data. Chocolate and light were administrated at time 0. All data are expressed as mean±SE. §, P<0.05 in comparison with the corresponding time point of MCh for chocolate or IL for light; *, P<0.05 in comparison with basal levels.

A maximal increase of prefrontal NE release was observed in control animals in response to both rewarding and aversive highly salient stimuli (WCh and IPL), and in stressed animals exposed to rewarding or aversive mildly salient stimuli (Fig. 1). WCh and IPL produced a time-dependent increase in NE outflow in the mpFC reaching a maximal increase of 60% at 120 min and 65% at 60 min, respectively. In stressed groups, MCh and IL produced a time-dependent increase of norepinephrine outflow, which reached a maximal increase of 70% at 120 min and 60% at 60, respectively. Finally, MCh and IL induced, in control groups, lower prefrontal norepinephrine release than in other groups (Fig. 1). Selective prefrontal NE depletion abolished increased NE release in all groups, irrespective of control or stress condition (Fig. 1). The average basal values of NE in the mpFC for each group did not differ significantly (Sham group, 1.27±0.17 pg per 20 µl; NE depleted group, 1.24±0.15 pg per 20 µl).

**Figure 2:**
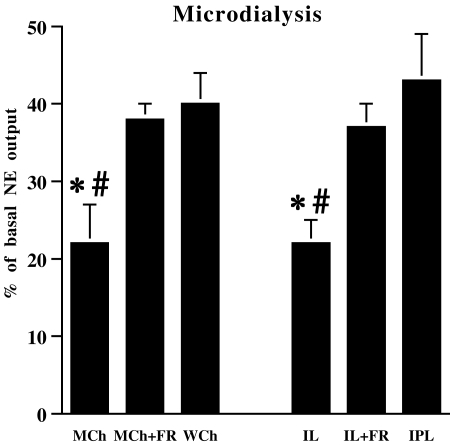
Overall mean percent increase of norepinephrine in medial prefrontal cortex (mpFC) induced by rewarding and aversive stimuli. Extracellular norepinephrine (NE) in mpFC of Sham animals exposed to chocolate (1 g) or light (milk chocolate in control, MCh; milk chocolate in food restricted, MCh+FR; white chocolate in control, WCh; intermittent light in control, IL; intermittent light in food restricted, IL+FR; intermittent-pulsating light in control, IPL). Results are the sum of time points (n = 8 for chocolate and 6 for light experiments) expressed as percent changes from basal levels (point 0) of each experimental group (*n* = 8–9 per group). All data are expressed as mean±SE. *, P<0.05 in comparison with the MCh+FR group for chocolate or the IL+FR group for light; #, P<0.05 in comparison with the WCh group for chocolate or the IPL group for light.

These results show that both appetitive and aversive stimuli induced graded NE release in the mpFC. Moreover, they indicate that the salience of unconditioned rewarding or aversive natural stimuli, graded by modulating the intrinsic properties of the stimulus or the motivational state of the organism, is directly related to the increase of prefrontal NE outflow. This conclusion is further supported by a data analysis carried out on overall mean percent values in Sham animals showing that MCh or IL in food restricted and WCh or IPL in control mice produce higher prefrontal NE release than MCh or IL in control mice (one-way ANOVA: Chocolate: F (2, 22) = 5.36, P<0.05; Light: F (2,16) = 5.17, P<0.05) (Fig. 2). Thus, taken together, data by microdialysis experiments indicate that prefrontal NE release is an index of the salience of stimuli.

### Effects of selective prefrontal norepinephrine depletion on place conditioning

We hypothesized that prefrontal cortical NE transmission plays a crucial role exclusively in motivational salience attribution to high salience-related stimuli. To investigate whether NE prefrontal transmission is necessary for acquiring conditioned appetitive and aversive properties to stimuli paired with highly salient primary rewards or aversive events, we assessed the effects of selective prefrontal NE depletion on place conditioning induced by both highly and mildly salient rewarding or aversive natural stimuli used in previous experiments.

**Figure 3:**
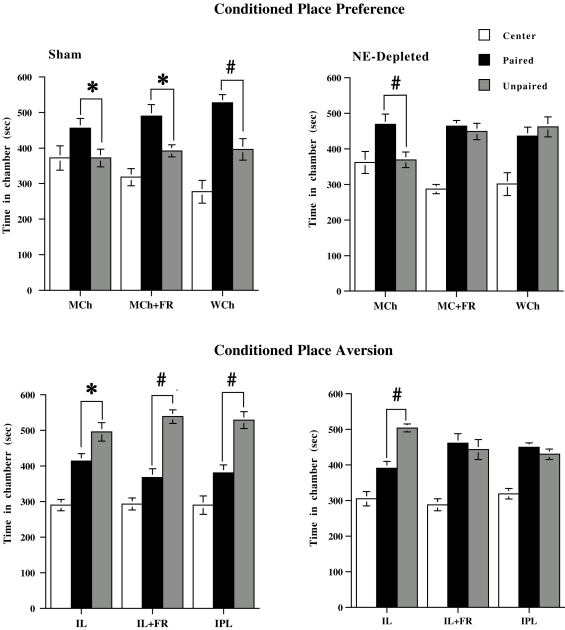
Prefrontal cortical norepinephrine depletion on place conditioning. Effects of prefrontal cortical norepinephrine depletion on conditioned place preference (upper panel) induced by chocolate (milk chocolate in control, MCh; milk chocolate in food restricted MCh+FR; white chocolate in control, WCh) and conditioned place aversion (lower panel) induced by light (intermittent light in control, IL; intermittent light in food restricted, IL+FR; intermittent-pulsating light in control; IPL). (Sham, norepinephrine-depleted (NE Depleted)). All data are expressed as mean (sec±SE) time spent in Center, Paired, and Unpaired chambers (*n* = 8 per group). * P<0.05 in comparison with the Unpaired chamber; # P<0.005 in comparison with the Unpaired chamber.

Selective prefrontal NE depletion abolished place conditioning induced by the highly salient stimuli, WCh and IPL, in control groups, as well as by the mildly salient stimuli, MCh and IL, in stressed groups. Thus, control animals showed a significant preference for the WCh-paired chamber (CPP) and a significant aversion to the IPL-paired chamber (CPA) (Fig. 3). Similarly, both control and stressed animals showed a significant preference for the MCh-paired chamber and a significant aversion to the IL- paired chamber (Fig. 3). Prefrontal NE depletion abolished CPP for WCh in control animals and for MCh in stressed animals, as well as CPA for IPL in control groups and for IL in stressed groups. Thus, NE-depleted animals in these groups showed no preference for, or aversion to, either chamber. However, no significant effect of depletion was evident in preference for and aversion to MCh and IL, respectively, in control animals (Fig. 3), thus indicating that prefrontal NE transmission determines motivational salience attribution to the to-be-conditioned stimulus only when the salience of the unconditioned stimulus is intrinsically high, because of its properties (highly salient stimulus), or is perceived as such due to the organism's motivational state (stressed animals).

Food restriction increased locomotor activity (total mean distance moved±SEM) in both pretest and test sessions of place preference and in pretest session of place aversion, irrespective of pretreatment condition (Sham, NE depleted), whereas prefrontal NE depletion did not affect locomotion in either pretest or test session of CPP or CPA, thus ruling out the possibility that prefrontal NE depletion produces an unspecific effect on locomotion (see Materials and Methods).

**Figure 4:**
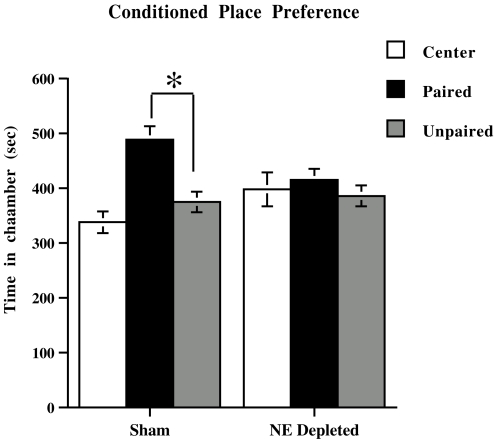
Prefrontal cortical norepinephrine depletion on place conditioning in isolated animals. Effects of milk chocolate consumption (1 g.) in place conditioning shown by Sham and norepinephrine-depleted (NE Depleted) animals submitted to social isolation. All data are expressed as mean (sec±SE) time spent in Center, Paired, and Unpaired chambers (*n* = 8 per group). *, P<0.05 in comparison with the Unpaired chamber.

Moreover, in additional experiments two other groups of mice (Sham, NE depleted) were subjected to different chronic stressful condition (social isolation) in order to assess whether the effect of prefrontal NE depletion on MCh-induced CPP can be ascribed to the homeostatic response to dietary deficiencies. Statistical results (see Materials and Methods) indicate that prefrontal NE depletion abolished CPP also in animals exposed to a chronic stressful condition other than food restriction, i.e., isolation (Fig. 4).

These results, together with those of microdialysis experiment, indicate that prefrontal NE transmission determines motivational salience attribution to the to-be-conditioned stimulus only under those conditions able to induce stronger increase in NE outflow in response to unconditioned stimulus, namely, when the motivational salience of the stimulus is high.

### Food Consumption

**Figure 5:**
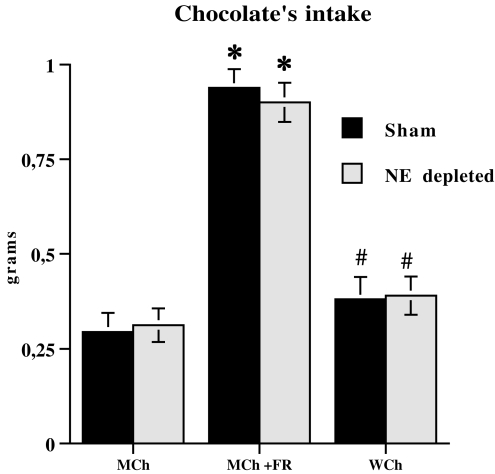
Chocolate consumption by Sham and NE depleted groups (milk chocolate in control, MCh; milk chocolate in food restricted MCh+FR; white chocolate in control, WCh) during microdialysis experiments. Data are expressed as total mean grams±SE. * P<0.005 in comparison with MCh group receiving the same pretreatment (Sham or NE depleted); # P<0.005 in comparison with the MCh+FR group receiving the same pretreatment (Sham or NE depleted).

Concerning chocolate consumption during microdialysis experiments, individual between-group comparisons showed that food restriction increased chocolate consumption irrespective of pretreatment condition (Sham, NE depleted) during microdialysis sessions. MCh+FR groups ate more chocolate than MCh and WCh groups, and there was a small, but non significant, difference in chocolate consumption between MCh and WCh mice. Finally, no significant difference was found between Sham and NE depleted groups for any treatment (MCh, MCH+FR, WCh) (Fig. 5).

**Figure 6:**
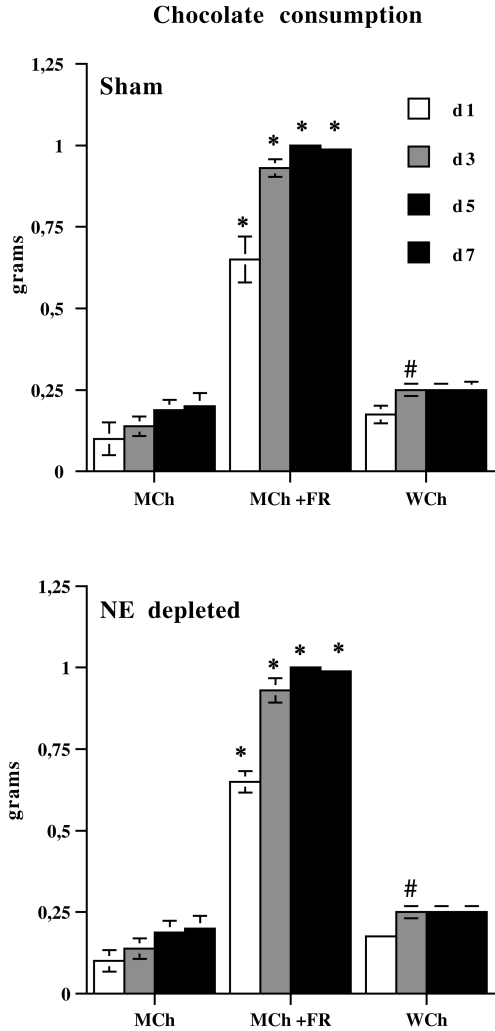
Chocolate consumption by Sham (upper panel) and NE depleted groups (lower panel) throughout conditioning procedure (day (d) 1, 3, 5 and 7 of pairing). Data are expressed as total mean grams±SE. * P<0.005 in comparison with the MCh and WCh groups; # P<0.05 in comparison with the MCh group.

Concerning chocolate consumption during conditioning phase of CPP, simple effects analyses for each time point revealed a strong significant difference between MCh+FR and the other groups (MCh, WCh) at all time points considered. Indeed, MCh+FR mice ate more chocolate on all conditioning days, irrespective of pretreatment (Sham, NE depleted), and a small significant difference was found between MCh and WCh at day 3. Finally, no significant difference was evident between Sham and NE depleted groups for any treatment (MCh, MCH+FR, WCh) (Fig. 6).

## Discussion

We assessed the hypothesis that prefrontal cortical NE transmission plays a crucial role exclusively in motivational salience attribution to stimuli related to highly salient events, irrespectively of valence. In order to demonstrate this, we assessed first whether the salience of unconditioned natural stimuli, modulated by their intrinsic properties as well as by the motivational state of the organism, produced a graded increase of prefrontal NE outflow. We report findings that explicitly link the level of prefrontal noradrenergic activation, as evaluated by intracerebral microdialysis, with the level of salience associated with both rewarding and aversive natural stimuli. A higher increase in prefrontal NE outflow was observed in response to highly salient stimuli, irrespective of their positive or negative valence, in comparison with mildly salient stimuli.

Moreover, previous exposure to a stressful condition increased the salience of mildly salient stimuli and led to enhanced prefrontal NE outflow comparable to that shown by animals exposed to highly salient stimuli. Thus, in sham control groups rewarding (WCh) and aversive (IPL) highly salient stimuli produced a large increase in prefrontal NE outflow, comparable to that produced by rewarding (MCh) and aversive (IL) mildly salient stimuli in sham-stressed animals. In addition, mildly salient stimuli (MCh and IL) induced a smaller increase of prefrontal NE release in sham control animals than in the other groups. Note that the major increase in prefrontal NE release shown by MCh+FR and WCh in comparison with MCh group cannot be ascribed to a difference in consumption patterns during the microdialysis session, because MCh+FR mice ate more chocolate than WCh and MCh, and only a small non significant difference in consumption was found between WCh and MCh. These data show no relationship between chocolate consumption and prefrontal NE release.

In fact, although MCh and WCh groups do not present significant differences in consummatory patterns (intake), it is nevertheless true that exposure to WCh induces stronger NE release than exposure to MCh, thus ruling out that NE release is related to intake and pointing, instead, to the salience of stimuli as a critical factor affecting NE release. Consequently, it is very unlikely that NE release in the mpFC of MCh+FR mice depends on higher intake.

This rules out the possibility that an increase in prefrontal NE outflow simply reflects amount and/or rate of chocolate consumption and, in fact, indicates that it reflects the salience of the stimulus. This conclusion is supported by results on aversive stimuli indicating that stress (FR) increases the release of NE in the mpFC of animals exposed to the mild aversive stimulus (IL+FR), thus increasing its salience.

Microdialysis experiments suggest that exposure to food restriction, which alters an organism's motivational state, increases the salience of both rewarding and aversive stimuli in the same way as that reported for numerous chronic stressors [2], [24], that is, by enhancing prefrontal cortical NE release.

Thus, our results, in agreement with previous reports, show that both unconditioned rewarding and aversive stimuli increase NE outflow within mpFC [14]–[17], [26]–[28]. Most importantly, however, they demonstrate that the increase in prefrontal noradrenergic release induced by both rewarding and aversive natural stimuli can be graduated on a continuum by varying the salience of stimuli.

Then, we provided evidence that prefrontal NE transmission is necessary for motivational salience attribution to both reward- and aversion-related stimuli only under those conditions able to induce stronger increases of NE outflow in response to highly salient unconditioned natural stimuli, independently of valence.

Results of place conditioning experiments indicate that prefrontal NE transmission determines motivational salience attribution to the to-be-conditioned stimulus only under those conditions able to induce a stronger increase of NE outflow in response to the unconditioned stimulus, namely, when stimulus salience is high. Thus, selective prefrontal NE depletion abolished the place conditioning induced by highly salient stimuli (WCh and IPL) in control groups and by mildly salient stimuli (MCh and IL) in stressed groups, but had no significant effects on the other groups. Behavior of NE depleted animals in MCh+FR, WCh, IL+FR and IPL groups was similar to that observed during the pretest session (data not shown), that is, they showed no preference/aversion for either compartment, thus indicating a random choice.

Note that prefrontal NE depletion did not interfere with either associative or mnemonic processes because, as previously shown, NE-depleted animals proved capable of learning a passive avoidance task [28] and of associating context with drug effects [27]. Moreover and most importantly, in the present experiment the same depletion did not affect the performance of control animals in CPP or CPA induced by mildly salient stimuli. In fact, both groups proved capable of associating the context with milk chocolate and intermittent light, that is, the natural mildly salient stimuli used in this study, thus indicating that effects of prefrontal NE depletion on CPP or CPA induced by highly salient stimuli, in present study, cannot be ascribed to unspecific associative or exploratory impairment.

It should be pointed out that the effect of prefrontal NE depletion on milk chocolate-induced CPP in food restricted mice cannot be ascribed to homeostatic response to dietary deficiencies because the same effect was present in intermittent light-induced CPA in food restricted animals as well as in milk chocolate-induced CPP in animals submitted to a different chronic stress, such as isolation. Moreover, concerning CPP experiments, data from chocolate consumption during conditioning phase rule out the possibility that observed effects can be ascribed to a difference in the consummatory pattern produced by prefrontal NE depletion. In fact, although MCh+FR ate more chocolate than WCh and MCh on all conditioning days and a small significant difference between WCh and MCh was evident at day 3 only, no significant difference was found between Sham and NE depleted groups for either treatment (MCh, MCH+FR, WCh).

Finally, data from total mean distance moved during pre-test or test sessions of place conditioning rule out the possibility that prefrontal NE depletion produces an unspecific effect on locomotion. Indeed, although FR groups (MCh+FR, IL+FR) showed greater locomotor activity than the other groups (MCh, WCh, IL, IPL) no significant difference in locomotor activity was evident between Sham and NE depleted groups in either pre-test or test sessions of CPP or CPA. Thus, it is conceivable that in NE depleted mice the lack of NE release, which was induced by exposure to highly salient stimuli in control animals and to mildly salient stimuli in stressed groups, prevented motivational salience attribution to the conditioned stimulus (spatial pattern) during the pairing sessions.

Thus, these results confirm our hypothesis and demonstrate that prefrontal cortical NE transmission is necessary for the acquisition of conditioned properties to stimuli paired with highly salient natural rewarding or aversive events in a place-conditioning procedure.

Motivational salience attribution is related to the salience of an unconditioned stimulus [2], [24]. Thus, the more salient an unconditioned stimulus the more likely a neutral (to-be-conditioned) stimulus will be associated with it through motivational salience attribution. Many different factors have major regulatory role in motivated behaviors, including the internal variables of the organism (i.e. motivational state, stress response) and stimulus properties (i.e. salience or intensity), both of which affect motivational salience attribution processes [21]. Moreover, emotional arousal induced by motivational stimuli has been suggested to increase the attention given to stimuli influencing both initial perceptual encoding and following the consolidation process [29], [30]. Our results are consistent with these views and show that highly salient stimuli selectively engage prefrontal NE transmission to determine motivational salience attribution.

It has been recently proposed that appetitive and aversive brain systems act in a “congruent manner for processes sensitive to affective intensity (salience) but not valence” [31], thus suggesting that a common neural system might be involved in processing stimuli salience, irrespectively of valence.

Moreover, arousing pleasant or aversive stimuli that elicit valence-specific responses have been suggested to enhance attention and memory formation through a common, valence-insensitive pathway [31] and prefrontal cortex has been involved in processing both rewarding and aversive stimuli [17], [32]–[34]. Our results extend previous findings showing that prefrontal cortical NE is a neural system sensitive to the affective intensity of stimuli, irrespectively of their valence.

Previously, we demonstrated that NE prefrontal transmission determines motivational salience attribution through modulation of dopamine in nucleus accumbens [17]. Therefore, the effects of prefrontal NE depletion on CPP and CPA observed in the present experiments could depend on the impaired response of prefrontal-accumbal catecholamine system. Moreover, since amygdala is involved in Pavlovian conditioning of emotional responses and plays a specific role in modulating memory for arousing experiences [30], [35], and given the complex anatomical and functional connections between this brain area and prefrontal cortex [36], [37], a role of the prefrontal cortex-amygdala system in the effects of the highly salient stimuli reported here must be considered [31].

Finally, results of control animals exposed to mildly salient stimuli strongly indicate that a different brain network, not involving NE in the mpFC, mediates acquisition of conditioned properties to stimuli paired with primary rewarding or aversive events in place conditioning induced by mildly salient stimuli. Nevertheless, further study is needed to elucidate this point.

In conclusion, our data demonstrate that prefrontal NE determines motivational salience attribution selectively when intense motivational salience is processed due to either the intrinsic properties of the stimuli or the motivational state of the organism (i.e. stress), in the latter case affecting “perceived” salience. This is reminiscent of different psychopathologies characterized by a breakdown of proper salience assignment and by excessively intense motivational salience attribution related to rewarding or aversion related stimuli/experiences [1]–[6]. To our knowledge, this is the first report of a direct link between noradrenergic prefrontal activation, salience of natural stimuli, and motivational salience attribution to stimuli. The possibility of graduating the salience of stimuli on a continuum and of linking different degrees of salience with different levels of prefrontal noradrenergic activation and with motivational salience attribution processes provides a basis for elucidating brain mechanisms underlying a variety of neuropsychiatric disorders related to a breakdown of proper salience assignments and for developing increasingly effective therapies.

## Materials and Methods

### Animals

Male mice of the inbred C57BL/6JIco (C57) strain (Charles River, Como, Italy), which are commonly used in neurobehavioral phenotyping, 8–9 weeks old at the time of the experiments, were housed as previously described and maintained in a 12 hr/12 hr light/dark cycle (light on between 07.00 a.m. and 07.00 p.m.) [17], [27], [28]. Each experimental group consisted of 6–9 animals. All animals were treated humanely in accordance with the principles expressed in the Declaration of Helsinki. All experiments were carried out in accordance with Italian national law (DL 116/92) on the use of animals for research based on the European Communities Council Directive (86/609/EEC, 24 November 1986).

### Drugs

Chloral hydrate, 6-hydroxydopamine (6-OHDA) and GBR 12909 (GBR), were purchased from Sigma (Sigma Aldrich, Milan, Italy). Chloral hydrate (350–450 mg/kg) and GBR (15 mg/Kg) were dissolved in saline (0.9% NaCl) and injected intraperitoneally (i.p.) in a volume of 10 ml/kg. 6-OHDA was dissolved in saline containing Na-metabisulphite (0.1 M).

### Rewarding and aversive stimuli

To assess if the appetitive stimuli used were provided of different salience, in a preliminary CPP experiment, one pattern was consistently paired with white chocolate (1 g., Paired chamber) and the other with milk chocolate (1 g., Unpaired chamber). A single piece (1 g) of either MCh or WCh was used for microdialysis and the same quantity for each place conditioning pairing.

To assess if the aversive stimuli used were provided of different salience, in a preliminary CPA experiment, one pattern was consistently paired with intermittent pulsating light (Paired chamber) and the other with intermittent light (Unpaired chamber).

A lamp (with a white, 8 W, cold light bulb, 400 Lumen, Osram Duluxstar, P.R.C., which created bright illumination) was placed 3 cm above the microdialysis or the pairing chamber of place conditioning apparatus; a timer allowed turning the light on or off at 20 sec intervals (Intermittent Light, IL). Moreover, to increase the aversive effect of lighting, a superimposed timer randomly turned off light seven times for 0.5 sec during each 20-sec lighting period (Intermittent Pulsating Light, IPL).

As chronic stressful condition, animals were placed on moderate food-restriction schedule [38], [39] 4 days before experiments started. Mice were assigned to different feeding regimen, namely either receiving food ad libitum (Controls) or subjected to food-restriction regimen (Stressed). In the food restricted conditions, food was delivered once daily (07.00 p.m.) in a quantity adjusted to induce a loss of 15% of the original body weight. In the control condition, food was given once daily (07.00 p.m.) in a quantity adjusted to exceed daily consumption (17 g). Microdialysis and place conditioning experiments were carried out in experimental sound-attenuated rooms indirectly lit by a standard lamp (60-W).

### Microdialysis

Animals were anesthetized with chloral hydrate (450 mg/Kg), mounted in a stereotaxic frame (David Kopf Instruments, Tujunga, CA) equipped with a mouse adapter and implanted unilaterally with a guide cannula (stainless steel, shaft OD 0.38 mm, Metalant AB, Stockholm, Sweden,) in the mpFC [17], [27], [28]. The 1 mm-long guide cannula was fixed with epoxy glue; dental cement was added for greater stability. The coordinates from bregma (measured according to Franklin and Paxinos's atlas) [40] were: +2.52 AP; 0.6 L. The probe (dialysis membrane length 2 mm, o.d. 0.24 mm, MAB 4 cuprophane microdialysis probe, Metalant AB) was introduced 24 hours before microdialysis experiments. Animals were lightly anesthetized with chloral hydrate (350 mg/kg) to facilitate manual insertion of the microdialysis probe into the guide cannula and were then returned to their home cages. The outlet and inlet probe tubing was protected by locally applied parafilm. The membranes were tested for in vitro recovery of NE (relative recovery (%): NE = 11.2±0.72; n = 15) on the day before use in order to verify recovery.

The microdialysis probe was connected to a CMA/100 pump (Carnegie Medicine Stockholm, Sweden) through PE-20 tubing and an ultra-low torque dual channel liquid swivel (Model 375/D/22QM, Instech Laboratories, Inc., Plymouth Meeting, PA) to allow free movement. Artificial CSF (147 mM NaCl, 1 mM MgCL, 1.2 mM CaCl_2_ and 4 mM KCl) was pumped through the dialysis probe at a constant flow rate of 2 µl/min. Experiments were carried out 22–24 h after probe placement. Each animal was placed in a circular cage furnished with microdialysis equipment (Instech Laboratories, Inc.) and with home cage bedding on the floor. Dialysis perfusion was started one hour later, at which time the mice were left undisturbed for approximately 2 h before baseline samples were collected. The mean concentration of the three samples collected immediately before treatment (less than 10% variation) was taken as basal concentration. Before microdialysis experiments, control mice (Sham, NE depleted) were assigned to one of the different treatments (MCh, WCh, IPL, IL); stressed mice (Sham, NE depleted) were assigned to MCh or IL. Regarding experiments with chocolate, consumption during microdialysis sessions was assessed by weighing left over chocolate at the end of the experiment.

Dialysate was collected every 20 minutes for 120 (for IL or IPL treatment) or 160 (for MCh or WCh treatment) minutes. Only data from mice with a correctly placed cannula are reported. Placements were judged by methylene blue staining. Twenty microliters of the dialysate samples were analyzed by high performance liquid chromatography (HPLC). The remaining 20 µl were kept for possible subsequent analysis. Concentrations (pg/20 µl) were not corrected for probe recovery. The HPLC system consisted of an Alliance (Waters Corporation, Milford, MA) system and a coulometric detector (ESA Model 5200A Coulochem II) provided with a conditioning cell (M 5021) and an analytical cell (M 5011). The conditioning cell was set at 400 mV, electrode 1 at 200 mV, and electrode 2 at −250 mV. A Nova-Pack C18 column (3.9×150 mm, Waters) maintained at 30°C was used. The flow rate was 1.1 ml/min. The mobile phase was as previously described [17], [28]. The assay detection limit was 0.1 pg.

### NE depletion in the mpFC

Anesthesia and surgical set are described in the preceding paragraph. Animals were injected with GBR (15 mg/Kg) 30 min before the 6-OHDA micro-injection in order to protect dopaminergic neurons. Bilateral injection of 6-OHDA (1.5 µg/0.1 µl/2 min for each side) was made into the mpFC (coordinates: +2.52 AP; ±0.6 L; −2.0 V with respect to bregma [40], through a stainless steel cannula (0.15 mm outer diameter, UNIMED, Switzerland), connected to a 1 µl syringe by a polyethylene tube and driven by a CMA/100 pump (NE depleted group). The cannula was left in place for an additional 2 min after the end of the infusion. Sham animals were subjected to the same treatment, but received intracerebral vehicle after GBR administration. Note that in previous experiments we observed no significant difference between Sham-treated and naïve animals in basal or pharmacological/natural stimuli-induced prefrontal NE or dopamine outflow or in CPP or CPA test [17], [28], [41], thus ruling out the action of GBR on observed effects in present experiments.

Animals were used for microdialysis or behavioral experiments 7 days after surgery.

NE and dopamine tissue levels in the mpFC were assessed, as previously described [17], [27], [28], to evaluate the extent of depletion.

### Behavioral Experiments

#### Place Conditioning

Behavioral experiments were performed using a place conditioning apparatus [17], [27], [28], [42]. The apparatus comprised two gray Plexiglas chambers (15×15×20 cm) and a central alley (15×5×20 cm). Two sliding doors (4×20 cm) connected the alley to the chambers. In each chamber two triangular parallelepipeds (5×5×20 cm) made of black Plexiglas and arranged in different patterns (however, always covering the surface of the chamber) were used as conditioned stimuli. Before conditioning, control mice (Sham, NE depleted) were assigned to one of the different treatments (MCh, WCh, IL, IPL), stressed mice (Sham, NE depleted) were assigned to MCh or IL. The training procedure for place conditioning has been described previously [17], [27], [28]. Briefly, on day 1 (pretest), mice were free to explore the entire apparatus for 20 min. During the following 8 days (conditioning phase) mice were confined daily for 40 min alternately in one of the two chambers.

For conditioned place preference with chocolate, one pattern was consistently paired with palatable food (WCh or MCh, 1 g., Paired chamber) and the other with standard food (mouse standard diet 1 g., Unpaired chamber). For conditioned place aversion, one pattern was consistently paired with the experimental light (IL or IPL, Paired chamber) and the other with standard chamber lighting (Unpaired chamber). Stressed animals were placed on a food-restriction schedule [38], [39] 4 days before conditioning started. This schedule was maintained throughout conditioning.

Moreover, in additional experiments two other groups of mice (Sham, NE depleted) were subjected to different chronic stressful condition (social isolation) in order to assess whether the effect of prefrontal NE depletion on MCh-induced CPP can be ascribed to the homeostatic response to dietary deficiencies.

For all place-conditioning experiments pairings were balanced so that for half of each experimental group the unconditioned stimulus (MCh, WCh, IL, IPL) was paired with one of the patterns and for half with the other. Testing for the expression of CPP or CPA was conducted on day 10 using the pretest procedure. Behavioral data were collected and analyzed by the ‘EthoVision’ (Noldus, The Netherlands), a fully automated video-tracking system [43]. The acquired digital signal was then processed by the software to extract two behavioral parameters: “distance moved” (in centimeters), as a measure of locomotion, and “time spent” (in seconds) in the compartments, which was used as raw data for preference/aversion scores in each sector of the apparatus of each subject [44]. Thus, data on locomotor activity were obtained from the same animals tested for CPP or CPA during pretest and test to rule out the possibility that prefrontal NE depletion produces unspecific effect on locomotion.

Finally, regarding conditioned place preference experiments, chocolate consumption was assessed by weighing left over chocolate at the end of each conditioning session (milk or white chocolate: on day 1, 3, 5 and 7).

### Statistics

#### Microdialysis

Statistical analyses were carried out on raw data (concentrations: pg/20 µl). The effects of prefrontal NE depletion on NE release in the mpFC of animals exposed to positive or aversive stimuli were analyzed by repeated measures ANOVA with two between factors (pretreatment, 2 levels: Sham, NE depleted; treatment, 3 levels: highly salient stimulus (WCh or IPL), mildly salient stimulus (MCh or IL), mildly salient stimulus plus stress (MCh+ FR or IL+FR), and one within factor (time, 9 levels: 0, 20, 40, 60, 80, 100, 120, 140, 160 for chocolate or 7 levels: 0, 20, 40, 60, 80, 100, 120 for light). Simple effects were assessed by one-way ANOVA for each time point. Individual between group comparisons were carried out when appropriate by post hoc test (Duncan Multiple Range Test).

Statistical analyses for the effects of rewarding stimuli on prefrontal NE outflow revealed a significant pretreatment×treatment interaction (F (2, 288) = 5.16, p<0.05). Simple effect analyses revealed a significant effect of time only for the Sham group and a significant difference between Sham and NE-depleted groups exposed to chocolate. Moreover, a significant effect of differently salient stimuli on prefrontal NE outflow was evident between MCh, MCh+FR and WCh.

Statistical analyses for the effects of aversive stimuli on prefrontal NE outflow revealed significant pretreatment×treatment interaction (F (2,192) = 3.64; p<0.05). Simple effect analyses revealed a significant effect of time only for the Sham groups and a significant difference between Sham and NE-depleted groups after light exposure. Moreover, a significant effect of stimuli salience on prefrontal NE outflow was evident between IL, IL+FR and IPL.

Finally, one-way ANOVA was carried out on percent values from basal levels in order to evaluate overall mean percent increase of NE in the mpFC of Sham groups induced by rewarding and aversive stimuli.

#### NE depletion in the mpFC

The effects of prefrontal NE depletion on tissue levels of dopamine and NE in the mpFC were analyzed by two-way ANOVA. The factors were as follows: pretreatment (2 levels: Sham, NE depleted), and experiment (2 levels: behavioral experiment, microdialysis experiments). Individual between-groups comparisons were carried out when appropriate by post hoc test (Duncan Multiple Range Test). Two-way ANOVA for the effects of prefrontal NE depletion on dopamine and NE tissue levels in the mpFC showed a significant pretreatment effect for NE only (F (1,189) = 2.23; p<0.0005) but no experimental effects. NE tissue levels were as follows: Sham group = 702±32; NE-depleted group = 71±13 ng/g wet tissue; Dopamine tissue levels: Sham group = 199±19; NE-depleted group = 189±16 ng/g wet tissue.

Moreover, it was previously shown that selective prefrontal NE depletion did not affect basal DA outflow nor DA outflow induced by pharmacological rewarding [27], [28] or natural aversive [41] stimuli in prefrontal cortex of mice or rat, thus showing that NE and DA release is independent.

### Behavioral experiments

*Place Conditioning*. For place conditioning experiments, statistical analyses were performed calculating the time (sec) spent in center (Center), experimental light/chocolate- (Paired) and standard room lighting/standard food-paired (Unpaired) chambers on the test day. For preliminary conditioned place preference with milk or white chocolate, Paired- chamber was identified as the one in which the white chocolate was received. For preliminary conditioned place aversion with intermittent pulsating or intermittent light, Paired- chamber was identified as the one in which the intermittent pulsating light was presented.

*Effects of selective prefrontal cortical NE depletion on Place Conditioning*. Data from place conditioning experiments were analyzed using repeated measure ANOVA with two between factor (pretreatment, 2 levels: Sham, NE depleted; treatment, 3 levels: highly salient stimulus (WCh or IPL), mildly salient stimulus (MCh or IL), mildly salient stimulus plus stress (MCh+FR or IL+FR)), and one within factor (pairing, 3 levels: Center, Paired, Unpaired). Moreover, because the important comparisons were those between Paired and Unpaired chambers, mean comparisons of time spent in these chambers were made using repeated measure ANOVA within each group. Three-way ANOVA revealed significant pretreatment×treatment×pairing interaction for CPP (F (4,84) = 2.53; p<0.05), and CPA (F (4,84) = 3.17; p<0.05). Repeated measure ANOVA within each group revealed a significant effect of the pairing factor for Sham animals exposed to MCh (F (1,14) = 5.12; p<0.05), MCh+FR (F (1,14) = 7.31; p<0.05), WCh (F (1,14) = 11.7; p<0.005), IL (F (1,14) = 5.2; p<0.05), IL+FR (F (1,14) = 30.7; p<0.005) or IPL (F (1,14) = 21.3; p<0.005) and for NE depleted animals exposed to MCh (F (1,14) = 11.3; p<0.005) or IL (F (1,14) = 26.3; p<0.005). Data by MCh-induced CPP in isolated mice were analyzed using repeated measure ANOVA with one between factor (pretreatment, 2 levels: Sham, NE depleted), and one within factor (pairing, 3 levels: Center, Paired, Unpaired). Repeated measure ANOVA revealed a significant pretreatment×pairing interaction (F (2, 28) = 1.95; p<0.05)). Mean comparisons, within each group, of time spent in Paired and Unpaired chambers revealed a significant effect of the pairing factor for Sham animals only (F (1,14) = 13.78; p<0.005).

Finally, concerning preliminary conditioned place preference with milk or white chocolate and conditioned place aversion with intermittent pulsating or intermittent light, data from WCh-induced CPP or IPL-induced CPA were analyzed by repeated measure ANOVA.Data concerning distance moved (total mean distance moved (centimeters)±SEM,) in whole apparatus during pretest or test sessions of CPP or CPA were analyzed by two-way ANOVA. Factors were the following: pretreatment (2 levels: Sham, NE depleted) and treatment (3 levels: highly salient stimulus (WCh or IPL), mildly salient stimulus (MCh or IL), mildly salient stimulus plus stress (MCh+FR or IL+FR)). Individual between-group comparisons were carried out when appropriate. Two-way ANOVA revealed no significant pretreatment×treatment interaction and a significant treatment main effect only for CPP pretest and test (pretest: F (2,42) = 31.7; p<0.005; test: F (2,42) = 3.28; p<0.05) and for CPA pretest (F (2,42) = 23.09; p<0.005), but no pretreatment effect.

#### Chocolate consumption

Data from chocolate consumption during microdialysis experiments were analyzed by two-way ANOVA. Factors were the following: pretreatment (2 levels: Sham, NE depleted) and treatment (3 levels: highly salient stimulus (WCh), mildly salient stimulus (MCh), mildly salient stimulus plus stress (MCh+FR)). Two-way ANOVA revealed no significant pretreatment×treatment interaction and only a significant treatment main effect (F (2,33) = 156.7; p<0.005). No significant difference was found between Sham and NE depleted groups for any treatment (MCh, MCH+FR, WCh).

Data from chocolate consumption during the conditioning phase of CPP were analyzed using repeated measure ANOVA with 2 between factors (pretreatment, 2 levels: Sham, NE depleted; and treatment, 3 levels: highly salient stimulus (WCh), mildly salient stimulus (MCh), mildly salient stimulus plus stress (MCh+FR)) and one within factor (pairing day: 1, 3, 5, 7 for milk or white chocolate). Simple effects were evaluated by one-way ANOVA for each time point. Repeated measure ANOVA revealed no significant pretreatment×treatment×pairing day interaction. However, significant treatment×pairing day interaction (F (6,126) = 14.01; p<0.005), treatment (F (2,126) = 1823.45; p<0.005) and pairing day (F (3,126) = 64.032; p<0.005) main effects were found.
